# Supplementary material for: Laser capture microscopy coupled with Smart-seq2 for precise spatial transcriptomic profiling
Source: Nat Commun. 2016 Jul 8;7:12139. doi: 10.1038/ncomms12139 (PMC4941116; doi:10.1038/ncomms12139)
Supplement: Supplementary Information — Supplementary Figures 1-8 and Supplementary Tables 1-2 [file ncomms12139-s1.pdf]

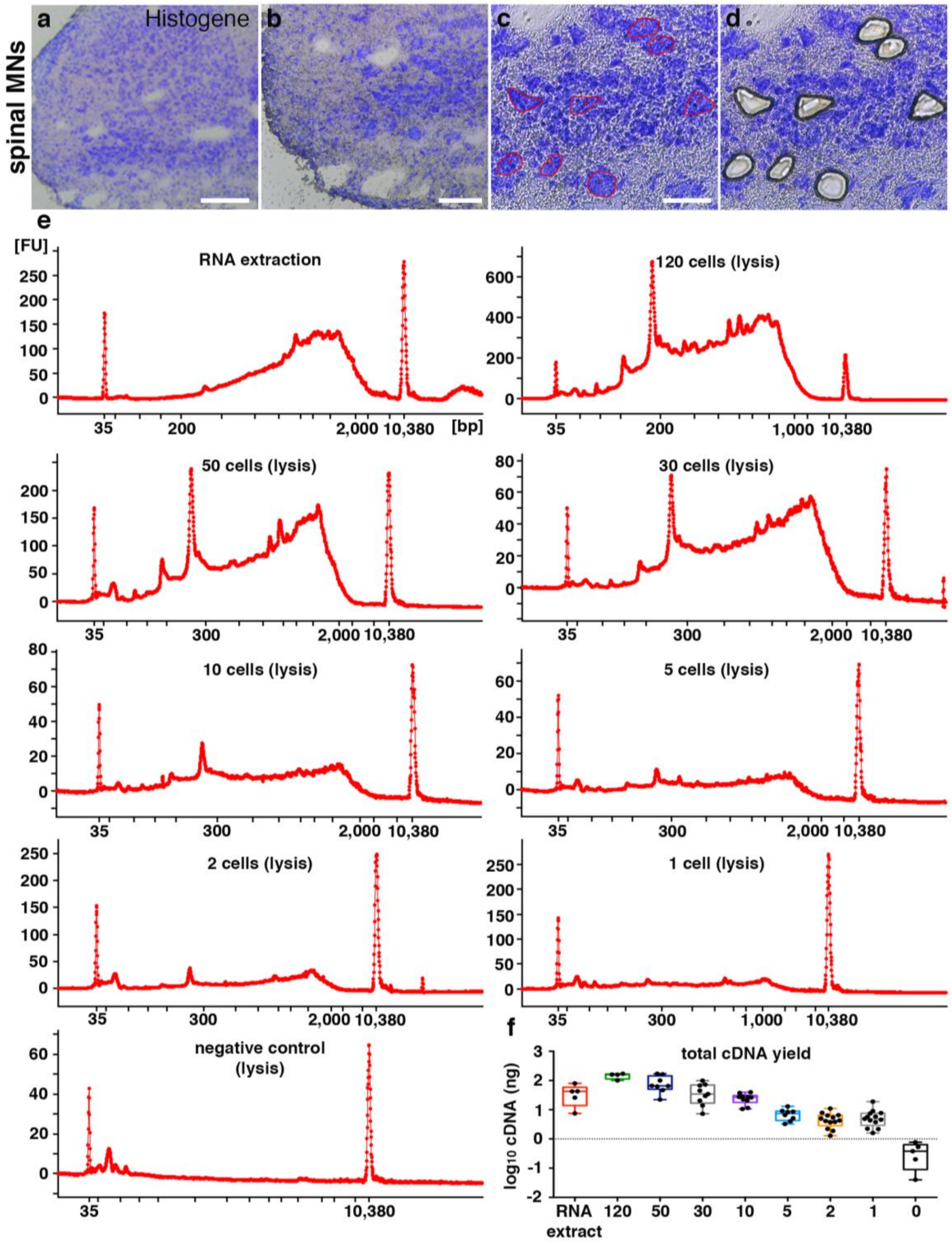

Supplementary Figure 1. Bioanalyzer profiles of LCM-seq cDNA libraries from mouse spinal motor neurons (MNs). (**a-d**) Spinal cords from postnatal day 5 mouse pups were sectioned at cervical and lumbar levels at 12 μm thickness. Histogene staining was used to visualize MNs, which were collected by laser capture microscopy (LCM) using a Leica

LMD7000 system. (**e**) Bioanalyzer profiles of cDNA libraries generated from 120, 50, 30, 10, 5, 2, and 1 cells using direct lysis were of comparable quality, as was the profile of cells subjected to RNA extraction prior to library construction. (**f**) The cDNA yield of 120 cells (adjusted *P*=0.0081, ANOVA Dunnett's multiple testing correction) subjected to direct lysis was significantly higher than that of the RNA extraction group, whereas 50, 30 and 10 cells resulted in a comparable cDNA yield (adjusted *P*=0.1611, *P*=0.9997 and *P*=0.9708, respectively; ANOVA, Dunnett's multiple testing correction with 'RNA extract' as the control group). Log_{10}-transformed data are shown as boxplot including individual values and with whiskers representing minimum and maximum. FU, fluorescent units; bp, basepairs. cDNA yield was measured in the range of 100 – 9,000 bp; scale bars: 200 μm in a, 100 μm in b and 50 μm in c (applicable to c and d).

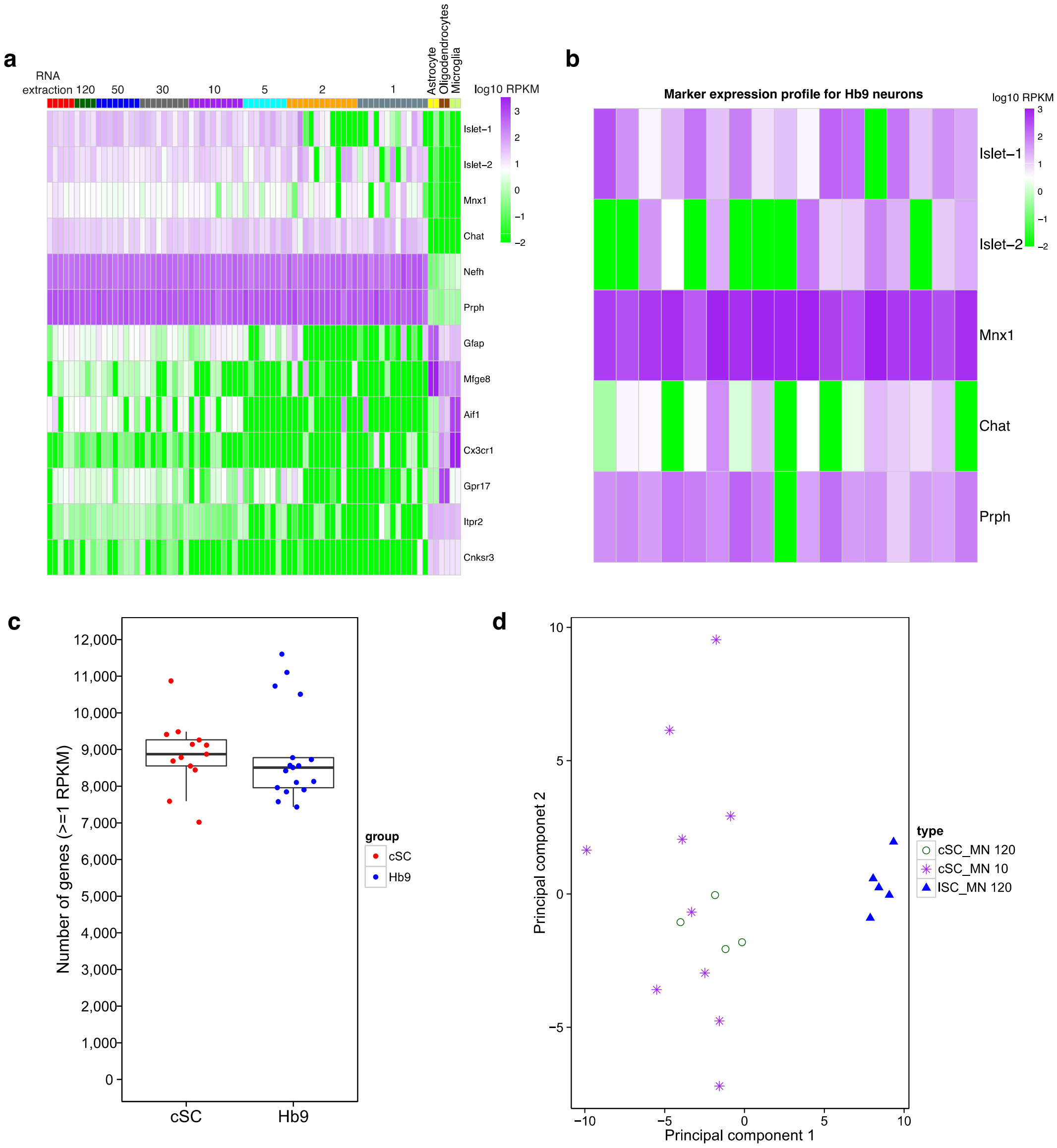

Supplementary Figure 2. Marker gene expression of spinal MNs across different samples. (a) LCM-seq libraries generated from RNA extraction and direct lysis of 120, 50, 30, 10, 5, 2, and 1 cells were analyzed for MN and glial marker gene expression and compared to a published set of glial populations (GEO Series accession number GSE52564). This analysis demonstrated LCM-isolated MNs expressed high levels of the MN markers *Islet-1/2*, *Mnx1* (Hb9), *Chat*, *Nefh* and *Prph*, while containing low levels of the glial markers *Gfap*, *Mfge8*, *Aif1*, *Cxcr1*, *Gpr17*, *Itpr2* and *Cnksr3*. Glial cells (astrocytes, oligodendrocytes and microglia) showed the opposite expression pattern. (**b**) Marker expression profile for 17 single live mESC-derived cSC Hb9^{+} (Mnx1) MNs. (**c**) A comparable number of genes were detected between live single mESC-derived MNs (n=17) and our single LCM-seq MNs (n=13) (*P*=0.5, Student's *t*-test (one-tailed)). (**d**) PCA of 120 and 10 cervical spinal MN

groups (cSC_MN_10 and cSC_MN_120, respectively) and 120 lumbar spinal MN groups (lSC_MN_120) showed that cervical MN groups clustered together.

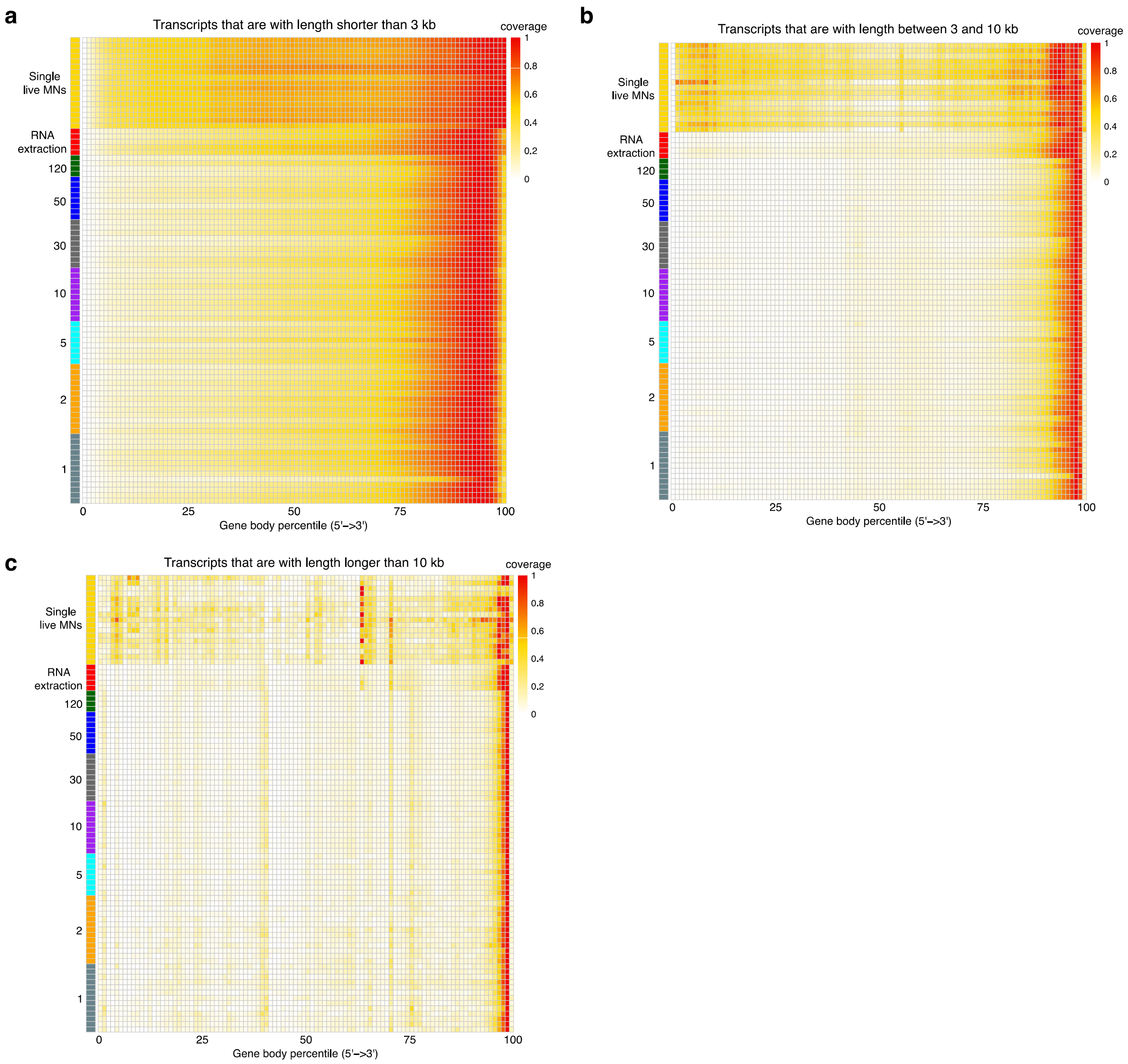

Supplementary Figure 3. Gene body coverage for transcripts with various ranges of lengths in different groups of samples. (**a**, **b** and **c**) are the gene body coverage for transcripts with length of < 3 kb, 3-10 kb and > 10 kb, respectively, in live single MNs and different cell numbers of mouse cSC groups. “Single MNs” refers to live mESC-derived single MNs sequenced by Smart-seq2. RNA extraction represents the groups of cSC neurons subjected to traditional RNA extraction method, while others are groups with different cell numbers of mouse cSC neurons subjected to the direct lysis approach.

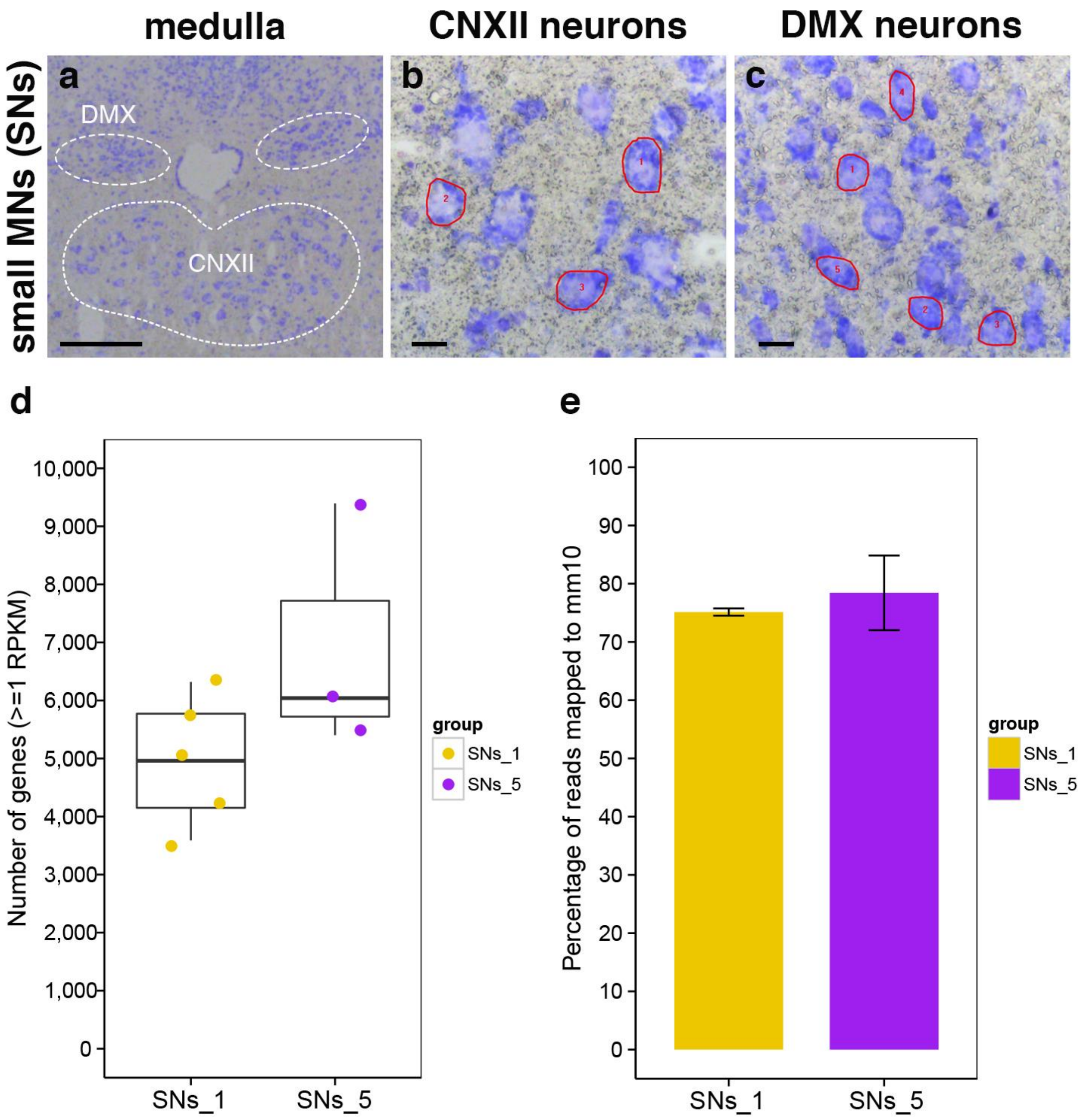

Supplementary Figure 4. LCM-seq of smaller motor neurons (SNs). (**a-c**) LCM-seq of SNs, single or pools of 5 cells, collected from the dorsal motor nucleus of the vagus nerve (DMX, area of the cells: 130-200 μm^2) and the hypoglossal nucleus (CNXII, area of the cells: 200-300 μm^2). (**d-e**) Analyses demonstrated that cells with smaller areas can be successfully sequenced using this method. (**d**) Single cell samples (n=5) contained an average of 4,958 detectable genes, while a mean of 6,945 detectable genes were identified in 5-cell samples (n=3). (**e**) The percentage of mapped reads were >70% and comparable between single and 5-cell groups (displayed as mean ± SEM). Scale bars: 200 μm in a and 15 μm in b and c.

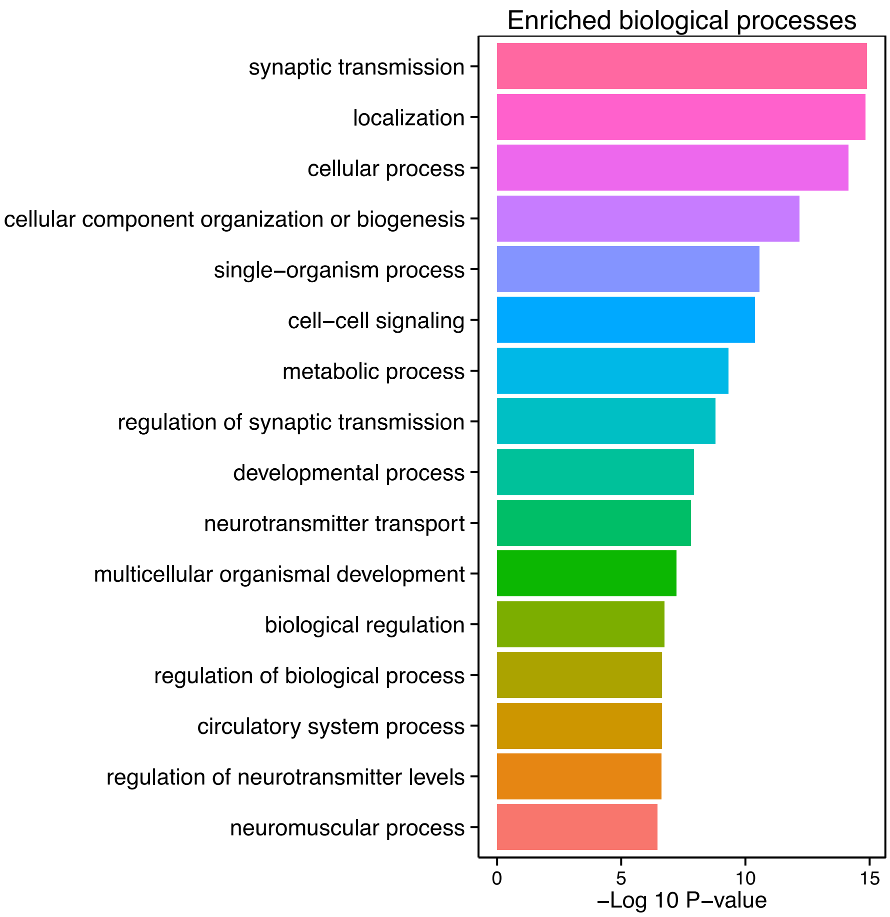

Supplementary Figure 5. Differential regulation of biological processes in cervical and lumbar spinal MNs. Using gene ontology (GO) enrichment analysis a number of processes with differential regulation between cervical and lumbar MNs were identified. Shown are the top 16 biological processes according to adjusted *P*-value (adjusted $P<0.01$, Fisher's exact test, topGO).

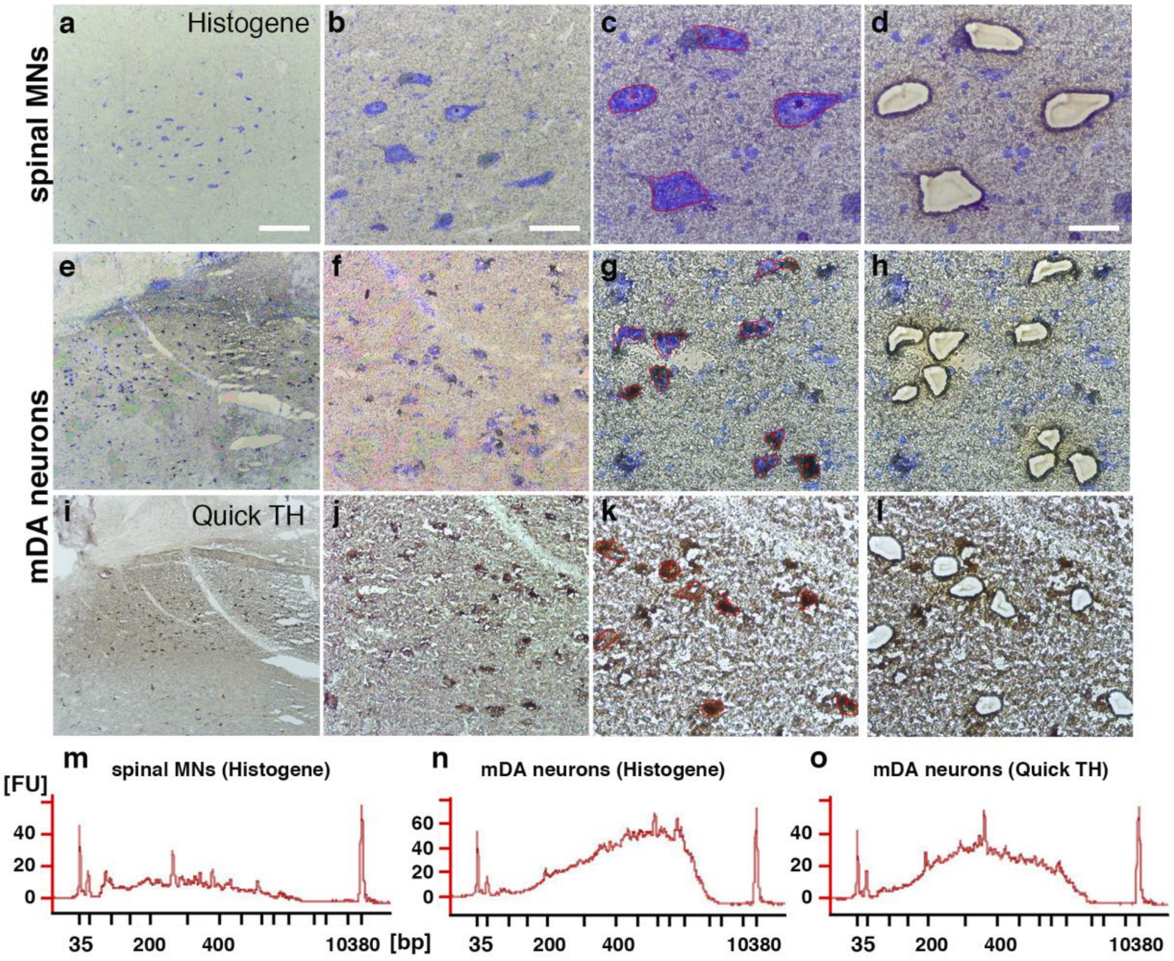

Supplementary Figure 6. LCM-seq was applied to human post mortem tissues to profile spinal MNs and midbrain dopamine (mDA) neurons. (**a-d**) Spinal MNs and (**e-h**) mDA neurons in human post mortem tissues were visualized using Histogene staining and isolated by LCM. (**i-l**) mDA neurons were also identified and collected based on a quick anti-tyrosine hydroxylase (TH) antibody staining. Bioanalyzer profiles showed that cDNA libraries could be successfully generated from (**m**) spinal MNs and (**n**) mDA neurons using Histogene, as well as from (**o**) mDA neurons using quick TH staining based on our LCM-seq protocol. FU, fluorescent units; bp, basepairs; scale bars: 400 μm in a (applicable to a, e and i), 200 μm in b (applicable to b, f and j) and 50 μm in d (applicable to c, d, g, h, k and l).

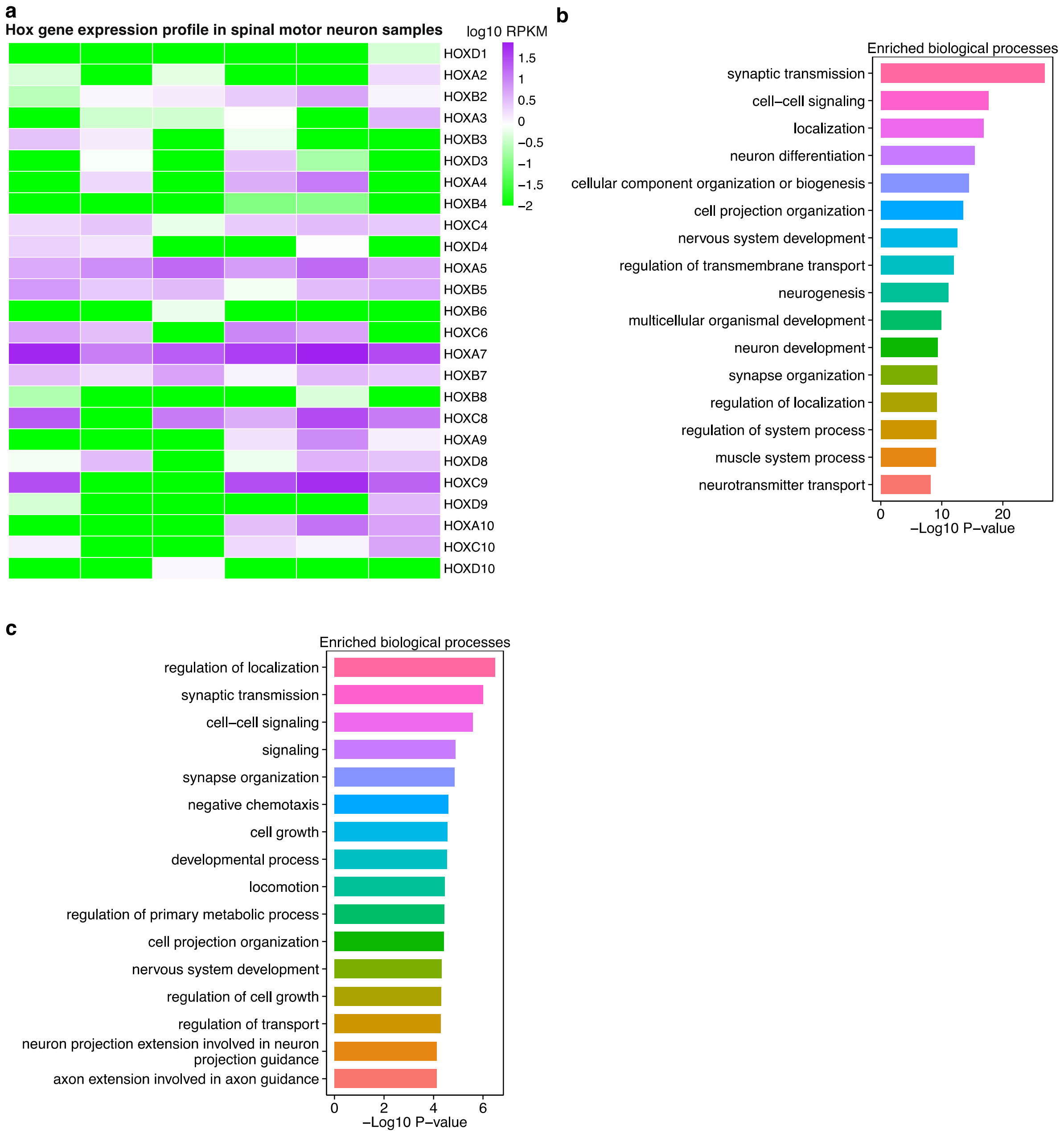

Supplementary Figure 7. Adult human spinal MNs maintained Hox gene expression and displayed a number of biological processes distinct from mDA neurons. (**a**) LCM-seq revealed that spinal MNs isolated from human post mortem tissues expressed a distinct set of Hox genes. (**b**) GO enrichment analysis showed that a number of enriched biological processes were identified for the differentially expressed genes between spinal MNs and mDA neurons isolated from human post mortem tissues. (**c**) Differentially expressed genes between SNc and VTA neurons were enriched in a number of biological processes. x-axis in b and c: adjusted *P*-value (adjusted $P<0.01$, Fisher's exact test, topGO).

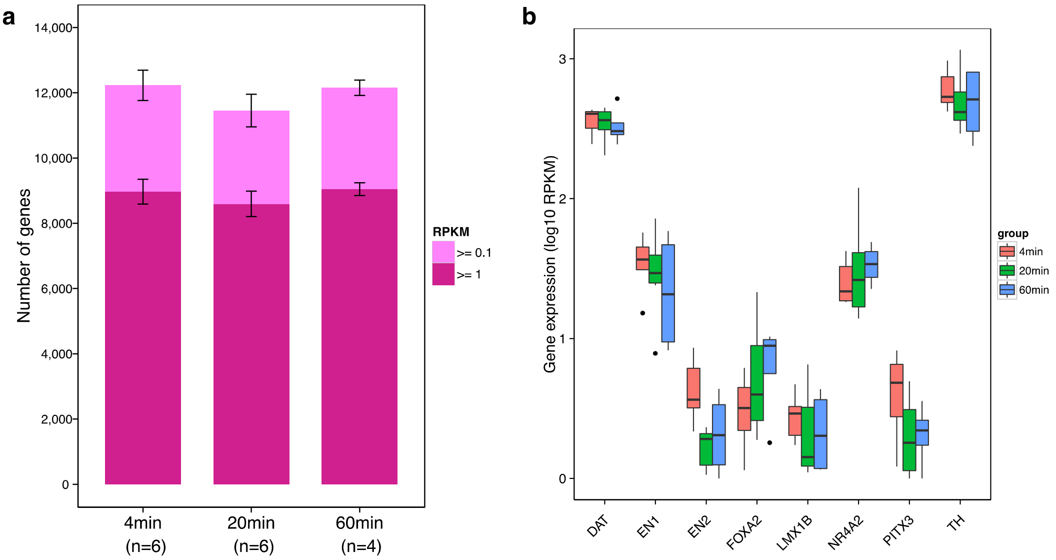

Supplementary Figure 8. Effects of increased antibody staining time on LCM-seq performance for human mDA neurons. (**a**) The number of detected genes remained comparable in human mDA neurons with increasing primary antibody incubation time, which ranged from 4 to 60 minutes (displayed as mean ± SEM). (**b**) mDA neuron marker gene expression remained unchanged when the primary antibody incubation time increased from 4, to 20 and 60 minutes. Boxes range from the 25th to the 75th percentile, and the centerline is the 50th percentile. Outliers are shown as dots.

Supplementary Tables

Supplementary Table 1. Characteristics of clinical cases used for LCM-seq

Case	Gender	Age	Post mortem time	Cause of death	Brain Bank	Targeted cells
1	F	68	3h	Pulmonary disease	NDRI	spinal MNs
2	F	71	7h 10m	Renal insufficiency	NBB	spinal MNs
3	F	72	7h 30m	Bile duct cancer	NDRI	spinal MNs
4	F	88	10h	Lung cancer	NDRI	spinal MNs
5	F	47	4h	Breast carcinoma	NBB	mDA neurons
6	F	50	4h 10m	Bronchocarcinoma	NBB	mDA neurons
7	F	52	6h 50m	Leiomyosarcoma	NBB	mDA neurons

F, female; MNs, motor neurons; mDA, midbrain dopamine

NBB, Netherlands Brain Bank, www.brainbank.nl

NDRI, National Disease Research Interchange, www.ndriresource.org

Supplementary Table 2. Details of human SNc samples stained with anti-tyrosine hydroxylase antibody for 4, 20 or 60 minutes and the corresponding LCM-seq libraries.

	N					
**Group**	**Cases**	**Samples**	**Cells isolated**	**Total area (μm^2)**	**cDNA yield (ng)**	**Total mapping ratio (%)**
**4 minutes**	3	6	75.5±1.3	24,867±769	5.5±0.9	81.1±1.8
**20 minutes**	3	6	75.0±0.6	30,290±1621	4.5±0.9	64.5±3.5
**60 minutes**	3	4	75.0±0.7	31,710±1872	4.1±0.5	62.4±6.3

SNc, *Substantia nigra compacta*; Values are mean ± SEM
